# Herbivorous fish feeding dynamics and energy expenditure on a coral reef: Insights from stereo‐video and AI‐driven 3D tracking

**DOI:** 10.1002/ece3.11070

**Published:** 2024-03-03

**Authors:** Julian Lilkendey, Cyril Barrelet, Jingjing Zhang, Michael Meares, Houssam Larbi, Gérard Subsol, Marc Chaumont, Armagan Sabetian

**Affiliations:** ^1^ School of Science Auckland University of Technology (AUT) Auckland New Zealand; ^2^ Leibniz Centre for Tropical Marine Research (ZMT) Bremen Germany; ^3^ Research‐Team ICAR, Laboratoire d'informatique, de robotique et de microélectronique de Montpellier (LIRMM), CNRS University of Montpellier Montpellier France; ^4^ The New Zealand Institute for Plant and Food Research Limited Auckland New Zealand; ^5^ University of Nîmes Nîmes France

**Keywords:** artificial intelligence, functional traits, metabolic traits, movement ecology, surgeonfish

## Abstract

Unveiling the intricate relationships between animal movement ecology, feeding behavior, and internal energy budgeting is crucial for a comprehensive understanding of ecosystem functioning, especially on coral reefs under significant anthropogenic stress. Here, herbivorous fishes play a vital role as mediators between algae growth and coral recruitment. Our research examines the feeding preferences, bite rates, inter‐bite distances, and foraging energy expenditure of the Brown surgeonfish (*Acanthurus nigrofuscus*) and the Yellowtail tang (*Zebrasoma xanthurum*) within the fish community on a Red Sea coral reef. To this end, we used advanced methods such as remote underwater stereo‐video, AI‐driven object recognition, species classification, and 3D tracking. Despite their comparatively low biomass, the two surgeonfish species significantly influence grazing pressure on the studied coral reef. *A. nigrofuscus* exhibits specialized feeding preferences and *Z. xanthurum* a more generalist approach, highlighting niche differentiation and their importance in maintaining reef ecosystem balance. Despite these differences in their foraging strategies, on a population level, both species achieve a similar level of energy efficiency. This study highlights the transformative potential of cutting‐edge technologies in revealing the functional feeding traits and energy utilization of keystone species. It facilitates the detailed mapping of energy seascapes, guiding targeted conservation efforts to enhance ecosystem health and biodiversity.

## Graphical abstract

Our research harnesses cutting‐edge technologies, including remote underwater stereo‐video and AI‐driven multi‐object tracking, to measure functional traits and the rates of energy expenditure of key grazing fish species on coral reefs. By doing so, we uncover the substantial influence of species like the Brown surgeonfish and Yellowtail tang on herbivore feeding dynamics, despite their comparatively low biomass. The potential of our approach lies in its ability to derive energy seascapes and novel ecosystem health indicators, offering a sophisticated toolset for enhancing conservation strategies and understanding the intricate balance of marine ecosystems.

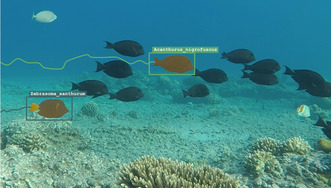

## 1 INTRODUCTION

The dynamics of herbivore consumption, both spatially and temporally, are pivotal in sustaining ecosystem functioning, particularly through their impact on energy and nutrient flows (Bauer & Hoye, 2014; Lundberg & Moberg, 2003). Influenced by resource availability, feeding preferences, and internal energy budgets, these dynamics shape herbivore foraging strategies (Gordon & Prins, 2019). Yet, a fundamental question remains in ecology: how do these factors interact to influence the expression of functional traits in herbivores, and what is their relative importance in this process (Bellwood et al., 2019)? Even further, behavioral adaptations driven by metabolic demands have far‐reaching implications for species interactions, community dynamics, and ecosystem functionality (Candolin & Rahman, 2023). Understanding these linkages is critical for predicting ecosystem responses to environmental changes and preserving biodiversity and ecosystem health (Schlägel et al., 2020).

To fully grasp the complexities of ecosystem functioning, going beyond studying foraging behavior is inevitable (Semmler et al., 2021). However, assessing metabolic traits and the energy invested into certain behaviors in the field (e.g. field metabolic rates) remains challenging – especially in aquatic organisms (Treberg et al., 2016). Synchronous consideration of functional traits and metabolic rates presents a promising approach to decipher the energetic foundations of species co‐existence and community interactions (Brandl et al., 2023). These traits are inextricably linked to an organism's strategy for acquiring, utilizing, and distributing energy, thus impacting its ecological fitness and shaping community functioning (Burton et al., 2011; Grémillet et al., 2018). Therefore, overcoming these challenges to measure energy expenditure (EE) in free‐roaming animals is crucial for assessing ecosystem‐level energy landscapes, enabling an understanding of metabolic constraints underlying animal movement and an ecosystem's ability to function (Shepard et al., 2013).

Through the use of remote underwater video (RUV), we can now further our understanding of aquatic herbivore fine‐scale feeding habits and their role in maintaining ecosystem balance (Lamb et al., 2020; Streit et al., 2019). Even further, with the rising application of RUV combined with advanced AI‐driven object recognition and tracking capabilities (Dell et al., 2014; Kays et al., 2015), our capacity to study animal behavior has improved considerably. Particularly in aquatic environments, remote underwater stereo‐video (RUSV) in combination with AI can meticulously track and analyze the 3D movements of foraging animals (Engel et al., 2021; Francisco et al., 2020). This innovative approach allows for a broader exploration of animal behavior, providing unprecedented insights into foraging strategies, feeding habits, and energy budgeting (Nathan et al., 2022). The resulting high‐resolution data becomes even more meaningful when combined with measurements of Overall Dynamic Body Acceleration (ODBA). This method assumes a direct correlation between an animal's movement and energy expenditure (EE), making it an effective proxy for estimating metabolic rates in free‐ranging animals (Gleiss et al., 2011; Gómez Laich et al., 2011). Indeed, previous research has successfully applied this method to study the relationship between field metabolic rates and fitness variations in wild animals (Grémillet et al., 2018).

Herbivorous fishes, characterized by diverse feeding‐related functional traits, substantially contribute to herbivory within coral reef ecosystems (Green & Bellwood, 2009; Kelly et al., 2016; Tebbett et al., 2020). These fishes play a key role in controlling the spread of epilithic algal turfs (EAT) and macroalgae fronds, which compete with coral colonies for light and space, facilitating the settlement of coral larvae and the eventual recovery of the reef (Ceccarelli et al., 2005; Roth et al., 2018). Disruptions to this intricate relationship could significantly impede the recovery process of these delicate ecosystems (Hoegh‐Guldberg et al., 2007; Pratchett, Hoey, & Wilson, 2014). Among herbivorous fishes, surgeonfishes are known for their ubiquitous presence and instrumental role in turf algae removal (Green & Bellwood, 2009; Kelly et al., 2016; Tebbett et al., 2020). A more in‐depth analysis of surgeonfishes' fine‐scale feeding behaviors is crucial to better understanding their role in reef resilience (Korzen et al., 2011).

In a coral reef ecosystem influenced by global changes, our study focuses on unraveling the community‐scale functional feeding traits, as well as feeding behaviors and EE of the two most dominant grazing herbivores, the Brown surgeonfish (*Acanthurus nigrofuscus*) and the Yellowtail tang (*Zebrasoma xanthurum*). Utilizing innovative tools such as RUSV and AI‐driven multi‐object tracking for measuring EE through allometric scaling and ODBA, our investigation aims to reveal the intricate feeding dynamics and energy utilization patterns of these functional key species. This approach is designed to enhance our understanding of their metabolic mechanisms and their roles within the broader reef community, contributing to the rapid assessment of field metabolic rates and the expression of functional traits in fish communities.

## 2 MATERIALS AND METHODS

### 2.1 Study site

Sampling was conducted on the reef located in front of the Inter‐University Institute for Marine Sciences (IUI) (29°30′7.0″ N, 34°55′3.7″ E) in Eilat, Gulf of Aqaba, between March 8 and 14, 2018. Prior to the 1970s, Eilat's reefs, nestled at the northern tip of the Gulf of Aqaba in the Red Sea, boasted exceptional within‐habitat coral species diversity, comparable to the Great Barrier Reef's reef flats (Loya, 2004). However, since the 1970s, these reefs have been under persistent anthropogenic stress, resulting in a worrying shift toward dominance by EAT, covering over 70% of available hard substrates (Bahartan et al., 2010; Loya, 2004). This sustained reef degradation has triggered a concerning drop in the region's marine biodiversity (Reverter et al., 2020).

### 2.2 Remote underwater stereo‐video surveys

To carry out the surveys, we deployed three calibrated stereo‐video systems, each comprising two GoPro cameras (four Hero 5 and two Hero 4), following the methodology outlined by Neuswanger et al. (2016). Footage was shot with a resolution of 1080p and a recording rate of 60 frames per second (fps). To validate the calibration accuracy of our stereo‐video systems, we measured the distances between dots on the front surface of the calibration frame (199 mm) across 10 different video frames and distances from the systems. The mean absolute errors (±SD) and mean absolute percentage errors for the three systems were 5.06 ± 5.79 mm and 2.5%, 6.80 ± 5.20 mm and 3.4%, and 5.73 ± 3.17 mm and 2.9%, respectively. Over three sampling days, we installed all three stereo‐video systems at a single sampling station each day, positioning them at depths of 2–3 m and approximately 10 m apart from each other.

For each system placement, sites were selected based on the diversity of grazable substrata, a key factor for understanding the varied feeding strategies of herbivorous reef fishes and categorizing different micro‐habitats (Green & Bellwood, 2009). This methodological choice facilitated a detailed analysis of the impact of substrata types on the foraging behaviors of these fishes within their respective ecological niches. Hence, sites with a heterogeneous mix of benthic substrate cover were preferred. Since surgeonfishes exhibit peak grazing rates around midday, the majority of our filming took place between 11:00 and 15:00 (Fouda & El‐Sayed, 1994; Montgomery et al., 1989). From nine rack placements, we obtained 13.5 h of analyzable video footage, with each original video lasting 1.75 h.

### 2.3 Assessment of benthic cover

At the start of each recording session, a 1 × 1 m quadrat was positioned in front of the cameras. To quantify the substrate cover within each quadrat, a long shot photograph was taken from above, capturing the entire quadrat in the frame. These images were uploaded to the program SketchAndCalc (iCalc Inc, Version 1.1.2), in which the 1 × 1 m quadrat was calibrated, so each transformed image contained roughly the same number of cells. This equated to ~1000 cells per image, each being around 5 cm^2^. The images with the canvas imprinted upon them were subsequently exported and annotated with each form of substratum – live coral and standing dead coral, bare calcium‐carbonate/sedimentary rock, coral rubble, and sand – having a corresponding color. We counted the annotated cells and calculated relative substrate cover (%).

### 2.4 Assessment of feeding dynamics

We manually measured fish total length (TL; mm) and functional traits, bite rate (bites min^−1^) as well as the distance between each consecutive bite (bite distance, in mm), only within the delimited quadrat area during the entirety of the recorded video footage. The initial 15 min of each video were discarded to allow for fishes to resume normal behavior after the quadrat was removed and divers left the site. The time at which a single fish entered the area of the quadrat to take bites from substrates until the time when it exited constituted a feeding event. For each feeding event, all bites were counted and then standardized against time to obtain bite rates.

We calculated individual fish mass according to each species' length‐weight relationship: mass = aTL^b^, where a and b for each species were informed from FishBase (see Table A1) (Froese et al., 2014). For each rack placement, fish biomass (g m^−2^) was calculated by adding all masses of individuals (from 19 species) that entered the quadrat during 45 min of filming to take bites. With this information, we were able to calculate total feeding rates (bites m^−2^ h^−1^) as well as feeding pressure as biomass standardized bites (kg bites m^−2^ h^−1^) per species (Longo et al., 2015).

The surgeonfishes *A. nigrofuscus* and *Z. xanthurum* contributed more than 86% of all recorded bites, and were thus selected as the model species to address our research question (Table 1, Videos 1 and 2). For each species, we manually recorded the substrate type for each bite observed in our stereo‐videos. This detailed data was then utilized to calculate Manly's feeding ratios, effectively illustrating the utilization of different substrate categories by individual fish in relation to the availability of these substrates across the reef (Manly et al., 2002). The summed feeding ratios per grazed substrate were compared to ascertain feeding preference (%) for the two target species across the entire reef (Pratchett, Hoey, Cvitanovic, et al., 2014). Further, for the focal species in each feeding event we averaged the distances between consecutive bites to obtain mean bite distance (mm). We conducted all manual measurements in the open source software VidSync Version 1.661 (Neuswanger et al., 2016).

**TABLE 1:** Replication details for the study on feeding behavior (feeding preferences and functional traits) and energy expenditure of *A. nigrofuscus* and *Z. xanthurum* on a coral reef in Eilat, Israel, Gulf of Aqaba. Energy expenditure assessments were not conducted simultaneously with the observation of feeding behaviors.

Species	Number of individuals (feeding behavior)	Number of replicates (feeding events)	Total bites	Number of trajectories (energy expenditure)
*Acanthurus nigrofuscus*	20	40	559	14
*Zebrazoma xanthurum*	10	72	1375	21

**VIDEO 1:** *Zebrazoma xanthurum* taking bites from the reef matrix on a coral reef in Eilat, Gulf of Aqaba, Red Sea.

**VIDEO 2:** *Acanthurus nigrofuscus* and *Ctenochaetus striatus* foraging on the reef matrix on a coral reef in Eilat, Gulf of Aqaba, Red Sea.

### 2.5 AI‐driven tracking of coral reef fish

In this study, we aimed to achieve AI‐driven automated fish detection, identification, and tracking from stereo‐video by performing several steps:

#### 2.5.1 Calibration

Our calibration process had to accurately estimate the 3D position of objects using our stereo camera system. This system captures two‐dimensional images, and our task was to project these onto a three‐dimensional plane. We employed the pinhole camera model for this purpose, a standard approach in photogrammetry, which facilitates the projection of 3D points onto the image plane via a perspective transformation.

However, pinhole cameras, like the ones we used in our study, are inherently prone to certain distortions. Radial and tangential distortions are common issues, which often result in straight lines in the real world appearing curved in the captured images. To address this, we first synchronized our stereo‐video image set in time through a clap. Following this, we recorded a checkboard pattern with both cameras, which is a standard practice in camera calibration.

**FIGURE 1:**
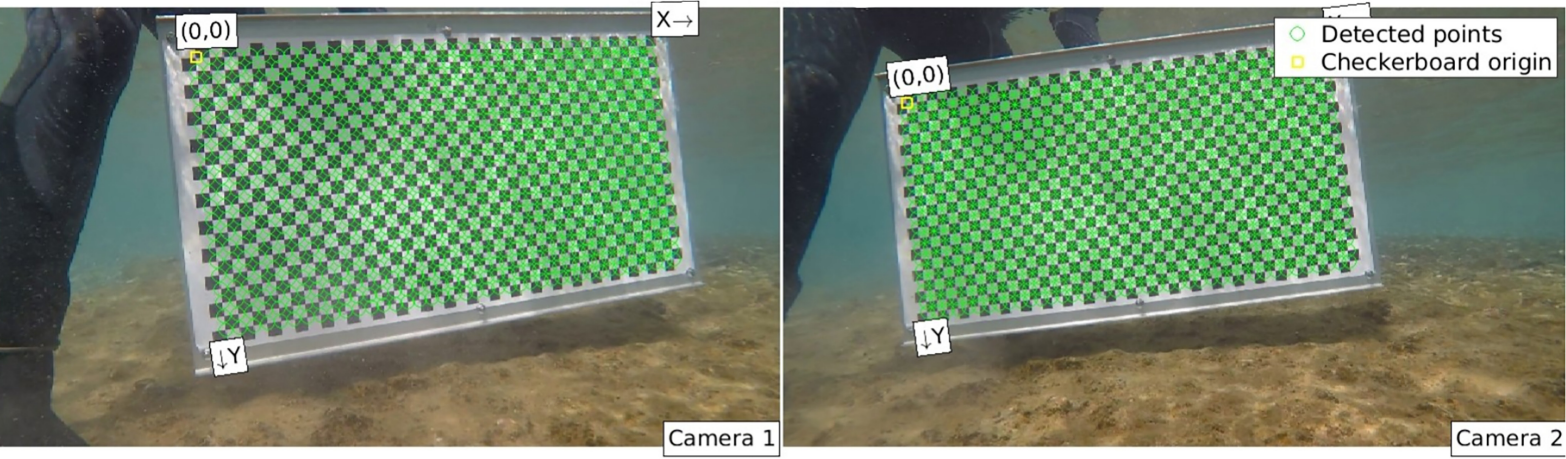
Detection of the checkboard pattern on the back of the calibration frame in Matlab.

For the actual calibration in Matlab (TheMathWorks, Version R2022a), we utilized Zhang's calibration method (Zhang, 2000). This method is particularly effective for correcting the mentioned distortions and aligning the 3D and 2D points accurately (Figure 1). The function allowed us to make use of the maximum number of rectangles from the calibration chessboard pattern on the back of our calibration frame. After calibrating our camera system in Matlab, we reformatted the detected image points and calibration parameters to align with OpenCV's data representation conventions for subsequent processing.

### 2.6 Stereorectification

**FIGURE 2:**
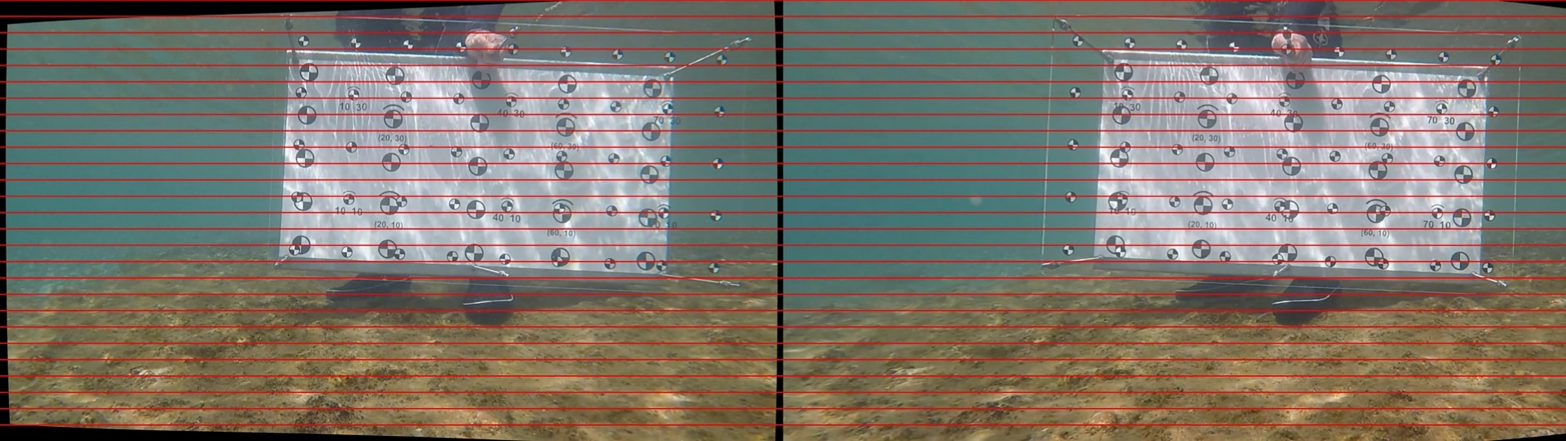
The calibration frame in a stereorectified frame pair in OpenCV.

Stereorectification aligns left and right camera images in such a way that they appear as if they have been shifted only horizontally. This alignment facilitates locating corresponding pixels in each image, which is crucial for accurately triangulating the depth of the scene. The rectification was done with OpenCV (Open Source Computer Vision Library, Version 4.9.0). The function CVstereoRectify takes the projection matrices and the distortion parameters of both cameras as input. As output, it provides two rotation matrices and two projection matrices in the new coordinates. We could now reassign all the pixels of the left image to the right image to get a rectified pair (Figure 2). Using this method of calibration, we obtained an overall mean [±SD] absolute re‐projection error of 0.9 [±1.9] mm which corresponds to 0.45% of the true value.

### 2.7 Object detection

For object detection, we employed the You Only Look Once Version 5 (YOLOv5) convolutional neural network (CNN) (Bochkovskiy et al., 2020). Initially, YOLOv5 was trained on a diverse dataset comprising 32,054 annotated images, covering 52 animal species, including corals, and divided into 80% training, 10% validation, and 10% testing sets, ensuring distinct locations for training and validation. To tailor YOLOv5 for our specific requirements, we retrained it with additional background images from the Red Sea videos, aiming for precise detection that differentiates fish from corals and other background elements. This retraining involved using images where fish were consistently present, treating moving foreground objects as noise, and calculating frame medians for background extraction.

**FIGURE 3:**
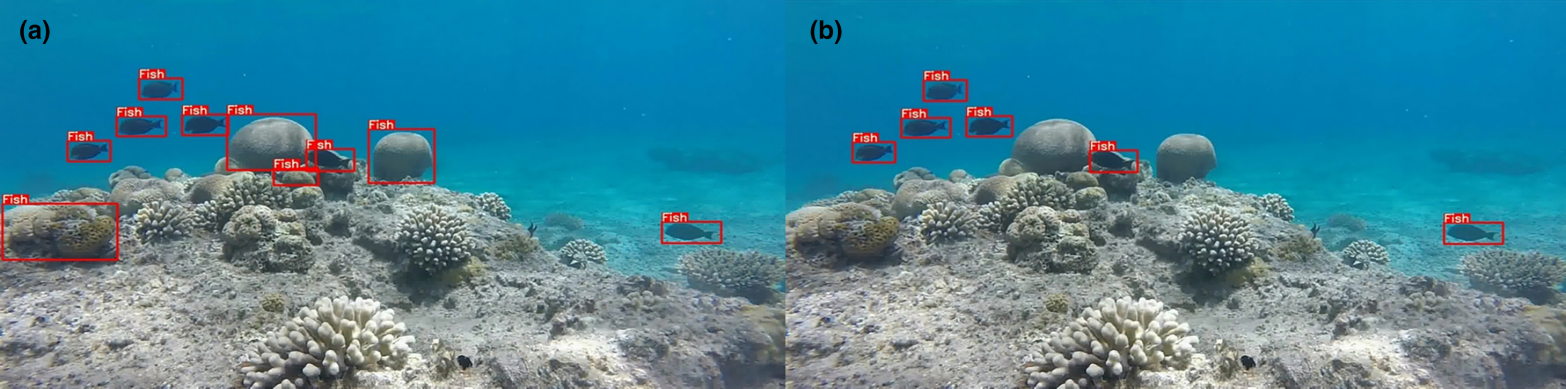
Performance of automatic object detection (a) before and (b) after background subtraction.

Our retraining strategy maintained the original data ratio while incorporating 10% of new background images. The goal was to achieve clear and accurate object detection, avoiding misidentification of non‐fish elements and reducing computational overhead. Post‐detection, the bounding boxes generated by YOLOv5 served as input for further classification and stereo matching. This step involved comparing feature vectors of bounding boxes from left and right camera images along the same epipolar line, ensuring accurate matching and spatial positioning of detected objects (Figure 3).

### 2.8 Object classification

We utilized iNaturalist (www.inaturalist.org), a social network and image repository used by community scientists globally, to classify detected fish species in our study (Shepley et al., 2021; Van Horn et al., 2018). iNaturalist serves as a platform for sharing biodiversity observations, where users contribute to the identification of various organisms. To augment training of our CNN, we selected research grade, location‐invariant images of identified fish species from this repository. Due to iNaturalist's limitations on mass image downloading, we employed web scraping techniques using the Beautiful Soup Python library (Richardson, 2007) and Selenium (ThoughtWorks, Version 4). To comply with the FAIR (Findable, Accessible, Interoperable, and Reusable) data principles, we made our training dataset openly available (Lilkendey, 2023): https://github.com/Knochenfisch/Functional‐and‐Metabolic‐Traits‐of‐Surgeonfishes/blob/6ece63aaaf20133084f8e74960796b37fd540317/data/iNaturalist_observations_training_dataset.csv

In the process of handling the high‐resolution images from iNaturalist, we first passed them through our detection system. This system primarily cropped the images to enhance focus on the subject animals. When multiple bounding boxes were detected in an image, suggesting the presence of various species, these instances required manual verification to ensure accuracy. Furthermore, we tackled the disparity in image resolution between our training dataset and the iNaturalist images. The iNaturalist images, being of a much higher resolution compared to the medium‐quality images our detector was trained on, were scaled down to match the resolution of our training dataset.

However, the iNaturalist dataset had limited images (*A. nigrofuscus*: 827, *Z. xanthurum*: 234), and therefore we employed transfer learning using weights computed from a previously recorded dataset from Mayotte, Indian Ocean, as a starting point (Villon et al., 2018). In this approach, we adapted a pre‐trained neural network model to our task, focusing on four specific classes. Since the feature extraction part of the model, represented by the early layers, was already trained on a large dataset for classifying fish species, we “froze” these layers to retain their learned general features. This decision was based on the similarity of the tasks – classifying fish species in both original and new contexts. We then modified the number of output nodes in the final layer from 52 to 4, tailoring it to our specific class requirements and enabling this layer to adapt to the nuances of our classification task. To account for the variability in iNaturalist images, caused by different capture conditions and sources, and to ensure the robustness and generalization of our method, we implemented a K‐fold cross‐validation strategy.

### 2.9 Multi‐object tracking

Finally, we implemented the DeepSORT framework – an enhanced version of the Simple Online and Realtime Tracking (SORT) algorithm – for multi‐object tracking (Wojke et al., 2017). More precisely, DeepSORT merges object detection with a tracker to follow and identify multiple targets in video sequences. It employs convolutional neural networks to extract visual features of objects and embeddings to represent unique identities, enabling precise association of objects across successive frames and handling occlusions. Additionally, DeepSORT integrates a Kalman filter on stereo‐video coordinates to correct misdetections and to display the bounding boxes continuously. This framework tracked each bounding box in both the left and right videos. Triangulation was performed to retrieve the 3D coordinates of the fish relative to the left camera, and we applied denoising to remove any erroneous data points. Overall, our approach enabled reliable and automatic object detection and tracking from stereo‐video (Video 3), providing valuable data for studying movements of the two focal species in their natural habitats. For comprehensive documentation on automated fish length measurements and the tracking algorithm, refer to Barrelet et al. (2023).

**VIDEO 3:** Coral reef fishes detected and tracked automatically through artificial intelligence while foraging on a coral reef in Eilat, Gulf of Aqaba, Red Sea.

To further optimize the quality of our 3D fish trajectory data, we implemented a systematic three‐step process in R (R Core team, Version 4.2.3):

1. *Outlier Removal*: Recognizing the sensitivity of Kalman filtering to outliers, due to its Gaussian‐distributed measurement noise assumption, we initiated our process with the interquartile range (IQR) method for outlier detection and removal. IQR is a statistical measure representing the range within which the middle 50% of data values lie, making it useful for assessing data variability and identifying outliers. We removed data points that fell below the lower bound (Q1–1.5 * IQR) and above the upper bound (Q3 + 1.5 * IQR) using a threshold of 1.5 times the IQR. This ensures that the filter operates optimally, delivering robust performance even when conditions deviate from the norm (Kassam & Poor, 1985; Kautz & Eskofier, 2015).
2. *Running Median Smoothing*: We used the zoo package to apply a 5‐point running median filter (Zeileis et al., 2023). The choice of a 5‐frame filter size, given our dataset's 60 Hz acquisition rate, adeptly balances noise reduction and the preservation of intrinsic data features, all while achieving our targeted minimum resolution of 10 Hz.
3. *Kalman Filtering*: Building upon the median‐smoothed data, we employed Kalman filtering, as suggested by Kalita and Lyakhov (2022). A Kalman filter is an algorithm that refines estimates of unknown variables over time using a series of measurements, even when these measurements contain noise or inaccuracies. It improves predictions by continuously updating them with new data (Welch, 1997). Kalman filtering was facilitated by the KFAS package, which hinges on the Gaussian distribution assumption of measurement noise (Helske, 2017).

### 2.10 Assessment of energy expenditure from 3D fish trajectories

To quantify EE we used change in velocity data obtained via the AI‐generated fish trajectories on the basis of stereo‐video footage (Krohn & Boisclair, 1994). We selected a subset of the longest detected surgeonfish trajectories, ensuring that the automatically measured surgeonfish individual TL fell within the length frequency distribution of each species determined manually via VidSync (Figure A2; Table A1).

From these trajectories, we computed velocity (cm s^−1^) by measuring the distances a fish moved between *X*, *Y*, and *Z* coordinates between consecutive video frames. Acceleration (cm s^−2^) was computed using the differences in velocity between consecutive frames. Using the methodology of Gleiss et al. (2011), we calculated ODBA, incorporating net acceleration to account for both movement and direction changes (Equation 1).

(1) $$\text{ODBA}=\left|\text{acceleration}\;X{\mathrm{cms}}^{-1}\right|+\left|\text{acceleration}\;Y{\mathrm{cms}}^{-1}\right|+\left|\text{acceleration}\;Z{\mathrm{cms}}^{-1}\right|$$

We implemented allometric scaling to correlate body mass with our ODBA data, as suggested by Chakravarty et al. (2023). To establish this correlation, we utilized Standard Metabolic Rate (SMR)‐mass relationships, which were derived from data recorded by Schiettekatte et al. (2022) (https://github.com/nschiett/activity_rate_fishes/blob/master/data/data_respirometry.csv). The correction factor K (mg O_2_^−1^ g^−E^ d^−1^) was derived from the intercept of the log–log regression of SMR against body mass, serving as a baseline metabolic rate per unit mass, essential for accurately scaling the EE calculations in relation to the specific body mass and activity levels of the studied species. The exponent E was obtained from the slope of the log–log regression line between log SMR and log mass. Our methodology involved using the functionally similar surrogate species, specifically *Ctenochaetus striatus* (SMR = 3.9994 × body mass^0.7789^) and *Zebrasoma scopas* (SMR = 3.9109 × body mass^0.6958^), as proxies for *A. nigrofuscus* and *Z. xanthurum*, respectively.

To account for variations in metabolic rates due to different ambient temperatures, we applied a temperature correction to our EE calculations. We employed the Q10 temperature coefficient, which quantifies the rate of metabolic change associated with a 10°C increase in body temperature. This factor is crucial for poikilotherms, as their body temperatures and metabolic rates can significantly vary with their thermal environment (Hill et al., 2012). We adopted a Q10 value of 1.92, typical for one of our surrogate species *Zebrasoma scopas* (McFarlane, 2016), and set our reference temperature at 28°C, aligning with the conditions under which Schiettekatte et al. (2022) conducted their metabolic studies in Mo′orea, French Polynesia. Our study temperature was selected as 21°C, representing the water temperature at sampling depth. The Temperature Adjustment Factor was calculated using Equation 2.

(2) $$\text{Temperature Adjustment Factor}=1.92^{\frac{28^{{}^{\circ}}C-21^{{}^{\circ}}C}{10}}$$

We converted metabolic rates to EE using a conversion factor of 14.1 J mg^−1^ O_2_ based on Brownscombe et al. (2017), with reference to the established bioenergetic standard for ammoniotelic animals (Elliott & Davison, 1975). For each frame, we computed EE (W) using Equation 3 where mass is in g and ODBA is unitless.

(3) $$\mathrm{EE}=\frac{K\times {\text{body mass}}^{E}\times \text{ODBA}\times \text{TemperatureAdjustmentFactor}\times 14.1{\mathrm{Jmg}}^{-1}O2}{60\times 60\times 24}$$

The culmination of this data processing protocol enabled calculations of mean velocity and EE for each recorded 3D trajectory: https://github.com/Knochenfisch/Functional‐and‐Metabolic‐Traits‐of‐Surgeonfishes/blob/6ece63aaaf20133084f8e74960796b37fd540317/output/3D_surgeonfish_trajectories.html

### 2.11 Statistical analysis

#### 2.11.1 Analysis of benthic cover composition and functional feeding traits

Spearman Rank Sum tests were utilized to identify correlations within the quadrat benthic cover composition. Also, analyses and visualizations of total bites, feeding rates, biomass and feeding pressure at the community level as well as surgeonfish feeding preferences were executed using JMP Pro (SAS Institute Inc, Version 16.0.0).

All following analyses were done in R. During initial data processing, outliers in our data on surgeonfish bites rates and inter bite distances were identified and excluded using the IQR method. Skewness in our data on bite distances and bite rates was rectified through a logarithmic transformation.

Six models were devised to assess the influence of “Species” and “Mass” on bite rates and bite distances. These models were designed to account for the potential non‐independence of observations:

1. A linear mixed‐effects model (LMM) with “Fish ID” and “Quadrat ID” as random effects, to compensate for resampling the same individual and the same quadrat, respectively.
2. Another LMM incorporating only “Fish ID” as a random effect, to address the potential non‐independence of observations from the same individual.
3. A basic linear model without random effects, to assess the direct effects of the fixed factors.

We employed the Akaike Information Criterion (AIC) to compare these models, favoring those with the best fit. Notably, linear models excluding random effects and using only “Species” as an explanatory variable consistently showed the lowest AIC values (Table A2). In our final models, homoscedasticity and normality of residuals were visually assessed using Residuals vs. Fitted Values and Quantile‐Quantile (Q‐Q) plots, respectively.

For exploring correlations between functional feeding traits (bite distance and bite rate) and environmental metrics, we employed LMMs. These models, developed with the lmer function in the lme4 package in R (Bates et al., 2015), included species as a fixed effect and individual fish and quadrat identity as nested random effects. Every substrate type was analyzed separately to avoid multicollinearity (Equation 4).

(4) Functional Feeding Trait∼Species ID+Substrate Type+(1|Fish ID:Quadrat ID)

#### 2.11.2 Analysis of velocity and energy expenditure

To ascertain significant differences in velocity and EE between the studied species, we employed Wilcoxon rank sum tests. Additionally, Levene's test was utilized to assess the equality of variances in the model residuals. We employed an Analysis of Covariance (ANCOVA) to investigate whether the slopes of the regression lines, depicting the relationship between EE and individual mass, exhibited significant differences between the two fish species.

We consulted the large language model ChatGPT (version 4.0, OpenAI) for two key aspects of our study. First, the model aided in refining our methodology, which involved integrating allometric scaling with ODBA data obtained from three‐dimensional trajectories. Second, ChatGPT provided assistance in English language editing, enhancing the clarity and coherence of the manuscript.

## 3 RESULTS

### 3.1 Benthic cover composition

**FIGURE A1:**
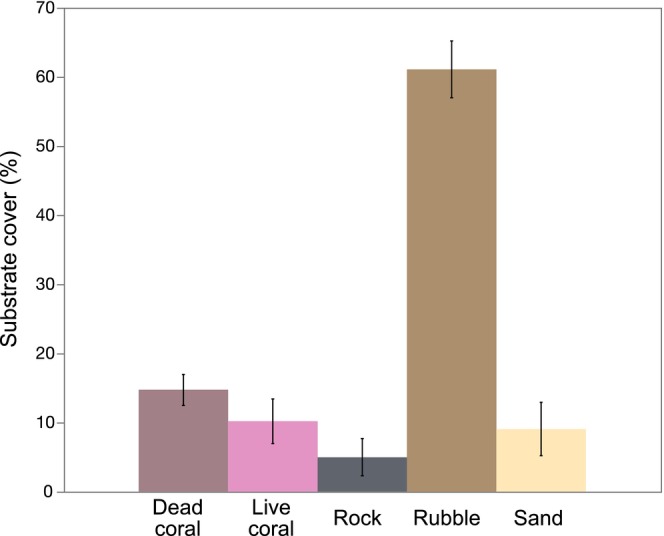
Relative benthic cover of substrates ± Standard Error across all 9 1 × 1 m study quadrats.

**FIGURE A2:**
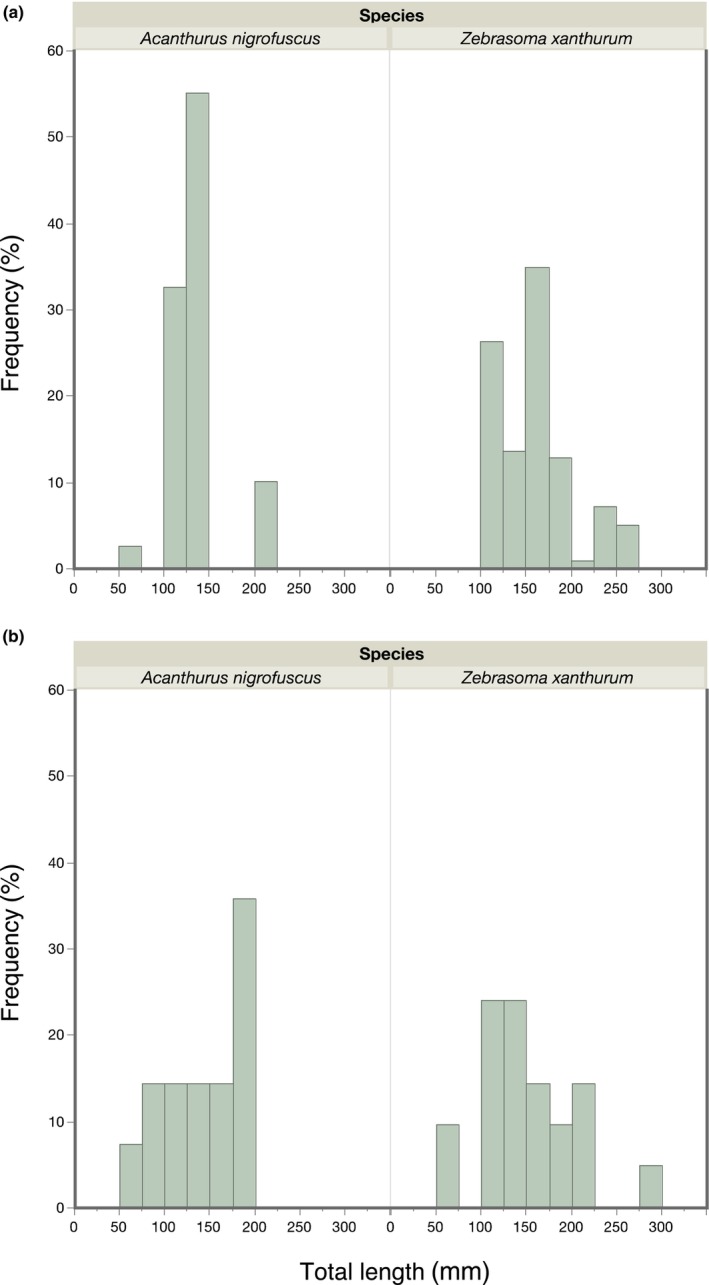
Total length frequencies of the focal species on a coral reef in Eilat, Gulf of Aqaba, measured manually using VidSync (a) and measured by object recognition driven by artificial intelligence in individuals used for this study (b).

The benthic cover of the study quadrats was dominated by rubble, followed by dead corals (Figure A1). Overall, only 10% of the substrate across all quadrats was covered by live coral. In the study quadrats, significant correlations were found within the substrate categories, where benthic cover in rock was negatively correlated with both standing dead coral (Spearman's *ρ* = −0.7667, *p* = .0159) and live coral (Spearman's *ρ* = −0.7000, *p* = .0358). Additionally, benthic cover in sand was positively correlated with rock (Spearman's *ρ* = 0.6833, *p* = .0424).

### 3.2 Community‐scale functional feeding traits

**FIGURE 4:**
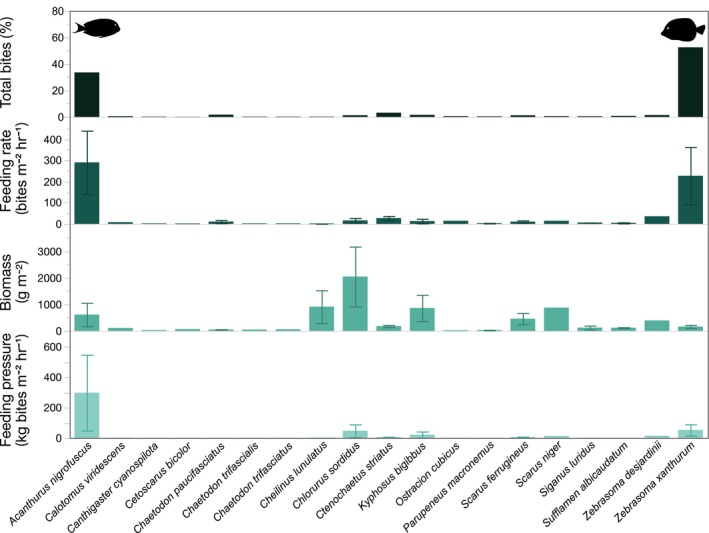
Percentage of total bites and mean (±Standard Error) feeding rate, biomass, and feeding pressure for all fish species recorded in 45 min of video per stereo‐video rack placement. Footage was obtained on a coral reef in Eilat, Gulf of Aqaba, Red Sea.

*Acanthurus nigrofuscus* accounted for 33.56% of total bites with a mean ± SD biomass of 616.57 ± 767.14 g, a mean feeding rate of 290.22 ± 259.07 bites m^−2^ h^−1^, and a mean feeding pressure of 298.61 ± 433.85 kg bites m^−2^ h^−1^. In contrast, *Z. xanthurum* contributed 52.52% of total bites, presenting a mean biomass of 166.53 ± 142.81 g, a mean feeding rate of 227.11 ± 331.15 bites m^−2^ h^−1^, and a mean feeding pressure of 52.78 ± 90.07 kg bites m^−2^ h^−1^. All other species recorded for this study contributed less substantially to total bites, feeding rates, biomass, and feeding pressure. Although certain species exhibited higher mean biomass values, such as Daisy parrotfish (*Chlorurus sordidus*) with a mean biomass of 2052.15 ± 1596.13 g and Broomtail wrasse (*Cheilinus lunulatus*) at 915.73 ± 870.09 g, their overall contributions to total bites, feeding rates, and feeding pressure were lower compared to the two focal species (Figure 4).

### 3.3 Surgeonfish feeding preferences and functional feeding traits

**FIGURE 5:**
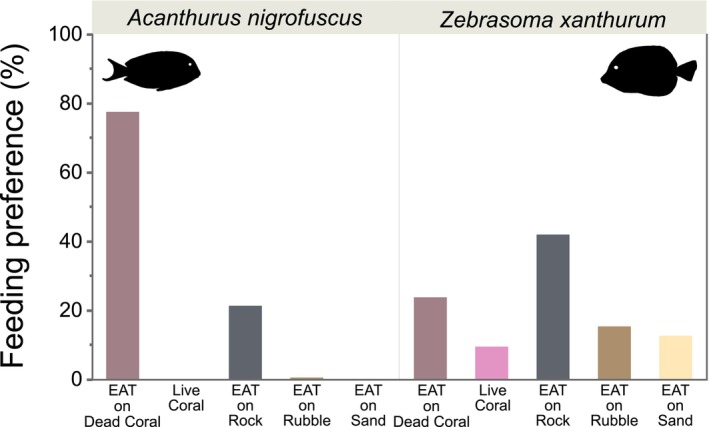
Feeding preferences of the two study surgeonfish species on a coral reef in Eilat, Gulf of Aqaba, Red Sea. EAT, epilithic algae turf.

In terms of grazed benthos, *A. nigrofuscus* primarily grazed EAT on standing dead coral, whereas feeding preference was generally more spread out across a range of substrates in *Z. xanthurum*, led by EAT on rock (Figure 5).

**FIGURE 6:**
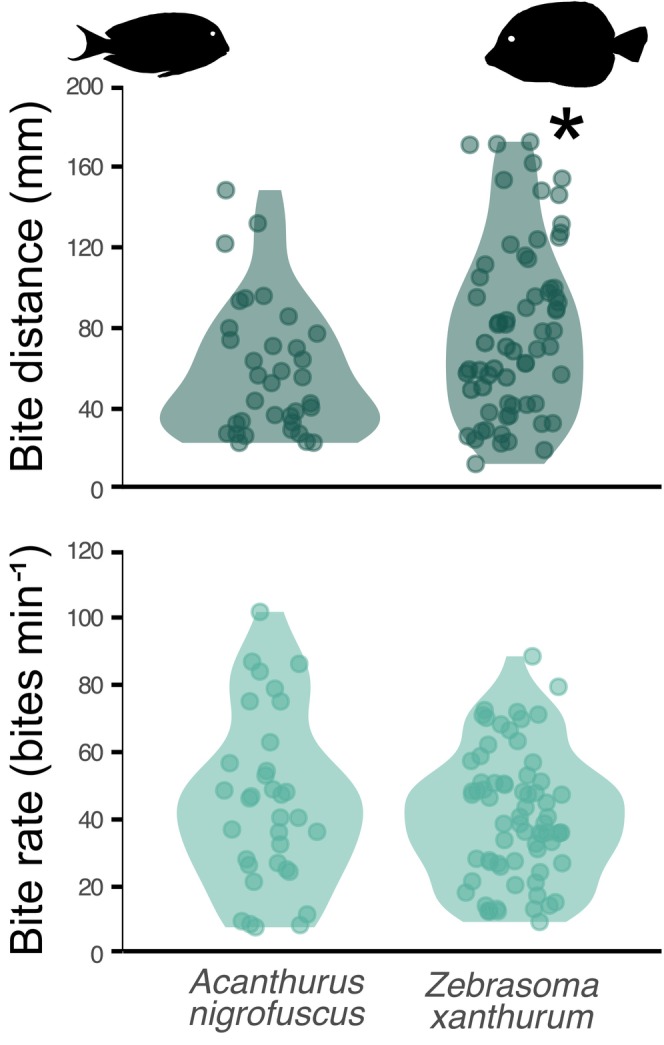
Violin plots of manually determined bite distances and bite rates of the two study surgeonfish species on a coral reef in Eilat, Gulf of Aqaba, Red Sea. The asterisk indicates a significant difference.

*Acanthurus nigrofuscus* exhibited a mean ± SD bite distance of 58.44 ± 32.54 mm and a bite rate of 44.82 ± 25.14 bites min^−1^. Conversely, individual *Z. xanthurum* exhibited an average bite distance of 79.52 ± 42.43 mm and a bite rate of 40.57 ± 19.08 bites min^−1^. The mean distances between consecutive bites were significantly greater for *Z. xanthurum* compared to *A. nigrofuscus*, as evidenced by the linear model (SE = 0.11914, *t* = 2.37, *p* = .0197) (Figure 6). Across both species, our data underscored a significant negative correlation between the percentage of sand cover and bite distance, estimating a decrease of 0.05 mm in the distance of consecutive bites for each percent increase in sand cover (LMM, SE = 0.01999, df = 17.89422, *t* = −2.225, *p* = .039).

### 3.4 Surgeonfish velocity and energy expenditure

**FIGURE 7:**
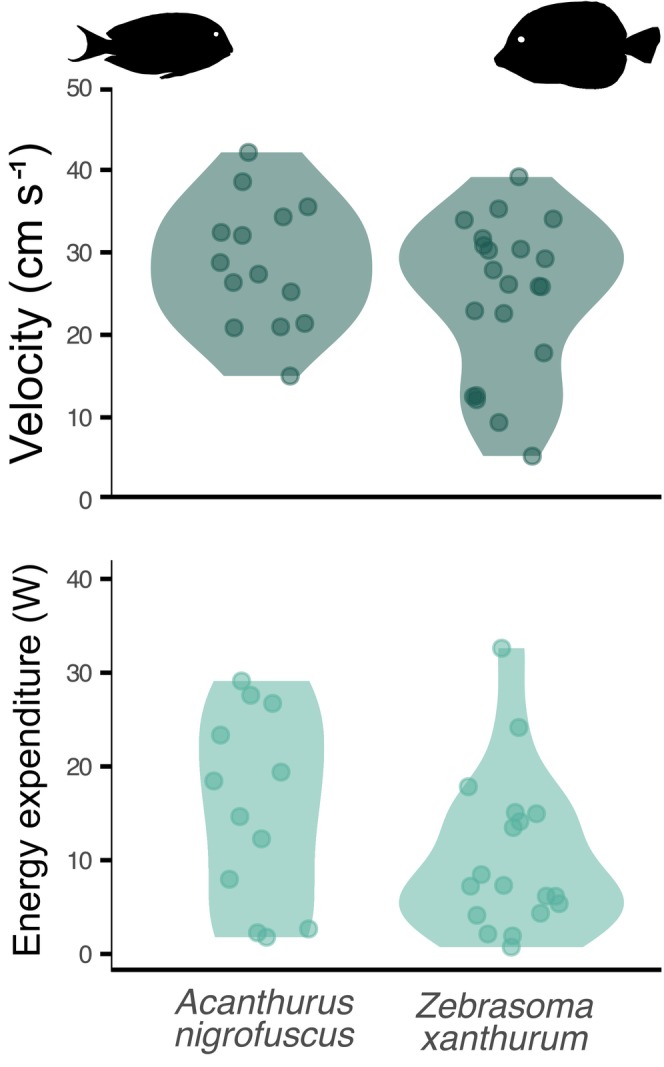
Violin plots showcasing mean velocities and rates of energy expenditures during foraging, based on artificial intelligence‐generated three‐dimensional fish trajectories for *Acanthurus nigrofuscus* and *Zebrasoma xanthurum*. Stereo‐video footage was captured in Eilat, Red Sea, Israel.

The mean ± SD classification results for the tracked individuals were 0.54 ± 0.09 in *A. nigrofuscus* and 0.78 ± 0.24 in *Z. xanthurum*. *A. nigrofuscus* exhibited a mean velocity of 28.6 ± 7.64 cm s^−1^, while *Z. xanthurum* displayed a mean velocity of 24.6 ± 9.46 cm s^−1^. Using the Wilcoxon rank sum test on individual mean velocities, the results showed no statistically significant difference between the two species (*W* = 179, *p* = .2931). In terms of rates of EE during foraging, *A. nigrofuscus* exhibited a mean EE of 21.12 ± 17.43 W, while *Z. xanthurum* had a mean EE of 19.95 ± 29.10 W. Upon applying the Wilcoxon rank sum test to individual mean EE values, a statistically significant difference was not identified in the mean EE between the two species (*W* = 179, *p* = .2931) (Figure 7). ANCOVA revealed that while mean mass significantly influenced EE (*F* = 57.59, *p* < .001), there was no significant difference in the slopes of the regression lines between the two surgeonfish species (*p* = .596), suggesting a consistent relationship between mass and EE across species.

## 4 DISCUSSION

Understanding the movement ecology and foraging behavior of herbivores is essential for insights into the functioning of anthropogenically stressed ecosystems like coral reefs. Herbivory plays a critical role in reef recovery (Eddy et al., 2021; Ledlie et al., 2007), yet our grasp of how species exhibit functional feeding traits in response to resource availability and metabolic constraints within these changing ecosystems remains limited (Goatley et al., 2016). By employing a novel methodology combining RUSV with AI‐driven 3D tracking, we established that *A. nigrofuscus* and *Z. xanthurum* are substantial contributors to grazing pressure on a Red Sea coral reef, despite their relatively low biomass. Our results reveal distinct foraging behaviors between the two species, characterized by variations in functional feeding traits, yet they maintain comparable rates of EE. This suggests that despite differences in their foraging strategies and interactions with the benthic environment, on a population level, both species achieve a similar level of energy efficiency. Such observations are pivotal in understanding the resilience and ecological strategies of herbivorous fishes within coral reef ecosystems. It underlines the complexity of the ecological roles played by different species and the importance of the interplay between energy budget and foraging behavior in maintaining the health and balance of coral reef systems.

### 4.1 Feeding dynamics of surgeonfish within the coral reef fish community

It is crucial to acknowledge and value the significance of herbivorous fish species that surpass predicted feeding pressure based on their biomass alone, as they play a vital role in maintaining ecosystem integrity (Longo et al., 2014). *A. nigrofuscus* alone accounted for over a third of the total bites observed, demonstrating substantial feeding activity despite its relatively moderate biomass when compared to other fishes on the reef for which we recorded bites. Similarly, *Z. xanthurum* contributed over half of the total bites, yet its mean biomass was significantly lower than most other species. Species‐specific mean feeding rates were comparable to surgeonfish feeding rates on Heron Island's near‐pristine shallow reefs (ca. 240 bites m^−2^ h^−1^), while feeding pressures in both species were markedly higher than on Heron Island (ca. 32 kg bites m^−2^ h^−1^) (Marshell & Mumby, 2015). It must be acknowledged that these metrics are not fully comparable as time of day and seasonality also affect grazing rates in herbivorous fishes (Ferreira et al., 1998; Magneville et al., 2023). However, our findings indicate that both *A. nigrofuscus* and *Z. xanthurum* play a disproportionately large role in grazing pressure relative to their biomass, underscoring their importance in maintaining ecosystem balance on our studied coral reef. Consistent with the findings of Paddack et al. (2006), our study underscores the pivotal role surgeonfishes play in mediating primary productivity in coral reef environments.

The foraging behavior of herbivores is often determined by various ambivalent and interrelated factors such as competition for resources, nutritional ecology, and physiology (Choat & Clements, 1998; Robertson & Gaines, 1986). In terms of foraging mode, *A. nigrofuscus* is using short nipping bites and spatulate teeth to remove algal matter from the EAT (Marshell & Mumby, 2012; Purcell & Bellwood, 1993; Tebbett et al., 2017). *Z. xanthurum*, on the other hand, is considered a browser, cutting off brown and red turf algae along the thallus (Fouda & El‐Sayed, 1994; Perevolotsky et al., 2020). In our study, *A. nigrofuscus* exhibited a specialized foraging preference for EAT on standing dead coral, while *Z. xanthurum* exhibited a more generalized grazing strategy, favoring a variety of substrates including EAT covering rock. *Z. xanthurum* is known to feed mainly on shallow rocks covered by turf algae (Perevolotsky et al., 2020), and species of the genus *Zebrasoma* have morphological features that enable them to feed in crevices and concealed locations to a much greater extent than other acanthurids (Brandl et al., 2015). These behaviors suggest niche differentiation at the microhabitat scale, potentially reducing competition and promoting coexistence within this coral reef ecosystems. Such a high spatial complementarity is often reported, even for seemingly similar functional groups and on disturbed coral reefs (Brandl et al., 2016; Brandl & Bellwood, 2014; Marshell & Mumby, 2012).

### 4.2 Surgeonfish functional feeding traits and rates of energy expenditure in relation to ecosystem functioning

Environmental changes, such as habitat alteration and climate shifts, significantly affect interactions within ecosystems, particularly between herbivores and their resources, due to changes in habitat structure and resource availability (Wong & Candolin, 2015). In our study, we observed a decrease in bite distances with an increase in the presence of sand, a less favorable foraging substrate. This variability in foraging behavior, triggered by resource scarcity, provides insight into how these species might respond to the ongoing degradation of coral reefs. Changes in how herbivores interact with their environment and express functional feeding traits can lead to cascading effects that ripple through the food web: These can manifest as top‐down effects, where alterations in consumer behaviors impact lower trophic levels, or as bottom‐up effects, where changes at lower trophic levels, such as the availability of food, influence higher trophic dynamics (Jochum et al., 2012; Pace et al., 1999). However, the complexity of species interactions within these networks makes it challenging to predict the full extent of these cascading processes and their ultimate impact on community structure and ecosystem functioning (Wong & Candolin, 2015).

Tracing the flow of energy plays a pivotal role in understanding ecosystem functioning, particularly in the context of coral reefs (Bellwood et al., 2019). The meticulous delineation of energy budgets provides important insights into the variations in fish fitness (Watson et al., 2020). For instance, variable metabolic rates, turnover rates of energy from food into usable biological energy, can impact interspecies competition, survival, and coexistence patterns on coral reefs (Clarke, 1989, 1992). Assessing metabolic traits of fishes can thus help in grasping complex, unpredictable outcomes in these species interaction networks (Brandl et al., 2023). In the context of our study on coral reef herbivores, EE serves as an indicator of the energy invested into foraging by two dominant grazing fish species. Longer bite distances in *Z. xanthurum* are an indication that the fish have to traverse longer distances to find feeding spots on a microhabitat scale. Despite differing foraging behaviors, the similar EE of these species suggests potential variations in diet nutritional quality or absorption efficiency (Clements et al., 2009; Schiettekatte et al., 2023). *Z. xanthurum* may also employ a more energy efficient biting physiology than *A. nigrofuscus* (Mihalitsis & Wainwright, 2024; Perevolotsky et al., 2020). Our results, therefore, provide direct insights into population‐level energy use strategies by investigating EE and the functional responses of fishes to changes in habitat quality – shedding light on the processes that mediate competitive interactions between the two model species (Brandl et al., 2023). Ultimately, combining approaches to assess energy flows across trophic levels and ecosystem scales will help to paint a more holistic picture of how energy moves through aquatic food webs (Robinson et al., 2023).

### 4.3 Automated tracking and inference of energy expenditure in fish

Recent AI advancements have significantly improved object recognition and tracking, leading to enhanced accuracy in species identification. However, there's a need for developing innovative, automated approaches and multidimensional data analysis in the fields of ecology and conservation (Besson et al., 2022; Nathan et al., 2022). Also, classification accuracy still heavily relies on the training data's quality and quantity (Muksit et al., 2022; Tan et al., 2022). Our automated method detects fish from stereo‐video images on a Red Sea coral reef using YOLOv5. Employing pre‐trained EfficientNet CNN and fine‐tuning with a limited dataset from iNaturalist, we automatically identify fish species, achieving an overall classification accuracy of 73% and showcasing transfer learning's potential. Our classification performance for one model species was suboptimal, potentially due to the close resemblance between *A. nigrofuscus* and *C. striatus*, which we also found on the reef, posing a challenge even for expert human observers. We expect that expanding the iNaturalist dataset with more varied images will enhance our system's ability to accurately classify these species. Our system, capable of tracking and classifying multiple objects, marks a significant advancement over previous studies lacking species identification (Engel et al., 2021; Francisco et al., 2020). Our methodology leveraged DeepLabv3 for the segmentation of fish within digital imagery, facilitating accurate measurements through 3D localization and triangulation techniques by pinpointing extremal points and leveraging Principal Component Analysis (Barrelet et al., 2023; Chen et al., 2018). In contrast, methodologies like the one presented by Coro and Walsh (2021) utilize YOLO for frame‐by‐frame detection, relying on image characteristics for size estimation without distance sensors which could lead to less precision in complex or overlapping scenarios. Our methodology employs DeepSORT for robust tracking, allowing for dynamic 3D trajectory creation thanks to triangulation. The presence of noise in our trajectory data led to inaccuracies, necessitating the use of denoising methods to enhance data quality. We adapted YOLOv5 and DeepSort for new environments and species, employing techniques like background subtraction and transfer learning, due to the scarcity of extensive training data. The model's effective adaptation from the Mayotte dataset to the Red Sea, despite requiring effort, underscores its flexibility and potential for wide‐ranging applications in marine environments.

Inference of EE in aquatic organisms remains a challenging task and metabolic studies conducted in respiratory chambers – if available at all – seldom capture complex activity patterns observed in the field (Treberg et al., 2016). Animal movement often involves variable acceleration patterns, and tracking acceleration has become a dependable way to study animal activity in the wild (Yoda et al., 2001). Moreover, measuring an animal's acceleration in all three dimensions provides a valuable proxy to infer EE while moving (Wilson et al., 2006). We are confident that our acceleration values are correct, as the swimming speeds for the two model species are well within the range of swimming speeds of other coral reef fishes observed using stereo‐video (Schiettekatte et al., 2022). We extrapolated EE from ODBA, calculated using AI‐generated trajectories, based on previously published relationships between SMR and mass from functionally similar species. This method aligns with approaches used in various studies, such as Gómez Laich et al. (2011) for Imperial Cormorants (*Phalacrocorax atriceps*) and Wright et al. (2014) for sea bass (*Dicentrarchus labrax*). Chakravarty et al. (2023) leveraged past allometric research to quantify EE in free‐roaming meerkats (*Suricata suricatta*) as a function of body size by deriving SMR‐mass relationships from a related species, the dwarf mongoose (*Helogale pervula*). Our EE values are consistent with studies that utilized acceleration data post‐calibration with respirometry in marine fishes (Brownscombe et al., 2017; Krohn & Boisclair, 1994), and are situated within the intermediate metabolic range between MMR and SMR of our surrogate species (Schiettekatte et al., 2022). This indicates the potential of combining accelerometry and allometry for estimating EE in aquatic species, especially when in‐lab calibration is not feasible.

### 4.4 Limitations and future research avenues

Future studies should overcome our research's limitations for a fuller understanding across varied marine environments. A notable constraint was our inability to measure bite distance, rate, and EE for individual *A. nigrofuscus* and *Z. xanthurum*, limiting our analysis to species‐level energy use strategies. Given the current reliance on labor‐intensive manual methods for evaluating functional traits, there's a pressing need for automated systems to identify and measure these traits accurately. This gap in methodology presents a significant avenue for future studies using methods that can integrate these crucial aspects of foraging behavior and metabolic activity in individual fish. Obtaining comprehensive data is crucial for understanding the relationship between feeding behavior and EE at an individual level. Additionally, the sample size in our study may not be large enough to represent the species' general behavior, potentially limiting our findings to the specific coral reef area we examined. Furthermore, integrating ecological variables like competition and predator–prey dynamics, along with direct SMR measurements, will contribute to a more nuanced understanding of herbivore foraging behaviors and energetics.

As AI and tracking technologies continue to advance, they will become integral to understanding ecological processes and ecosystem resilience (Besson et al., 2022). Incorporating AI into RUSV devices will revolutionize marine ecology research by streamlining data collection, improving methodological consistency, and expanding study scales, thereby elevating AI from a mere data recording tool to a fundamental aspect of ecological monitoring. Our approach offers potential for studying movement and energy budgets of keystone species across habitats and ecosystems, rapidly assessing metabolic traits in entire communities (Nathan et al., 2022). By analyzing acceleration patterns across communities, we can deduce “energy seascapes” in marine environments, mapping the varied energy costs of foraging in diverse settings (English et al., 2024; Wilson et al., 2012). These contributions are pivotal for developing ecosystem health indicators and shaping effective conservation strategies (Bograd et al., 2010). Such knowledge is invaluable for deriving targeted protection and restoration initiatives, thereby enhancing both biodiversity and overall ecosystem functionality.

## 5 CONCLUSIONS

In our study conducted on a Red Sea coral reef, we leveraged RUSV and AI‐generated 3D movement trajectories to delve into resource use patterns, the expression of functional feeding traits, and rate of EE – a key metabolic trait – in two dominant grazing fish species. Our innovative methodology revealed distinct foraging behaviors between two surgeonfish species, characterized by variations in functional feeding traits, yet they maintained comparable rates of EE. This suggests that despite differences in their foraging strategies and interactions with the benthic environment, on a population scale, both species achieve a similar level of energy efficiency. This study underscores the transformative potential of technologies like RUSV, AI‐driven fish identification, and 3D tracking in enhancing our understanding of metabolic traits and their role in big data‐driven conservation strategies. While our research was specific to a coral reef, it opens the door for further studies to explore ecological energetics and energy landscapes in various ecosystems.

## AUTHOR CONTRIBUTIONS

**Julian Lilkendey:** Conceptualization (lead); data curation (lead); formal analysis (lead); funding acquisition (lead); investigation (lead); methodology (lead); project administration (lead); resources (equal); supervision (equal); validation (lead); visualization (lead); writing – original draft (lead); writing – review and editing (lead). **Cyril Barrelet:** Data curation (equal); investigation (equal); methodology (equal); software (equal); supervision (equal); validation (equal). **Jingjing Zhang:** Formal analysis (equal); writing – review and editing (supporting). **Michael Meares:** Data curation (equal); formal analysis (supporting); investigation (equal). **Houssam Larbi:** Data curation (equal); formal analysis (supporting); investigation (equal); methodology (equal); software (equal); writing – original draft (supporting). **Gérard Subsol:** Methodology (equal); software (equal); supervision (equal); validation (equal). **Marc Chaumont:** Methodology (equal); project administration (equal); resources (equal); software (equal); supervision (equal); validation (equal). **Armagan Sabetian:** Conceptualization (equal); project administration (equal); resources (equal); supervision (equal); validation (equal); writing – original draft (equal); writing – review and editing (equal).

### OPEN RESEARCH BADGES

This article has earned Open Data and Open Materials badges.

## Data Availability

Scripts for automated detection of fish and measurement of their lengths, along with the multi‐object tracking algorithm, are available on GitHub (https://github.com/CBarrelet/Bubot). Our training dataset for species identification and the scripts used for the statistical analysis of functional feeding traits and metabolic traits, based on 3D fish trajectories generated by AI, are available on Github (https://github.com/Knochenfisch/Functional‐and‐Metabolic‐Traits‐of‐Surgeonfishes) and citeable through Zenodo (https://doi.org/10.5281/zenodo.10657695). The datasets generated during this study are available through PANGAEA (https://doi.org/10.1594/PANGAEA.957634).

## Appendix

**TABLE A1:** Mean (±Standard Deviation) total length (TL), bite distance, as well as *a* and *b* (from FishBase) of fish species recorded taking bites from the reef matrix in Eilat, Gulf of Aqaba, Red Sea.

Family	Species	TL (mm)	Bite distance (mm)	*a*	*b*
Acanthuridae	*Acanthurus nigrofuscus*	134.7 ± 30.1	66.0 ± 41.4	0.02455	2.97
	*Ctenochaetus striatus*	152.4 ± 16.6	123.4 ± 164.7	0.02344	3.06
	*Zebrasoma desjardinii*	202.9 ± 55.0	86.2 ± 4.6	0.02344	2.97
	*Zebrasoma xanthurum*	135.3 ± 28.1	81.7 ± 48.2	0.02344	2.96
Balistidae	*Sufflamen albicaudatum*	153.8 ± 10.7	246.9 ± 158.6	0.02570	2.94
Chaetodontidae	*Chaetodon paucifasciatus*	108.6 ± 17.6	294.8 ± 321.6	0.02291	3.00
	*Chaetodon trifascialis*	138.9	525.0	0.02138	2.95
	*Chaetodon trifasciatus*	130.8		0.02344	3.06
Kyphosidae	*Kyphosus bigibbus*	276.2 ± 51.0	197.0 ± 158.8	0.01660	2.98
Labridae	*Cheilinus lunulatus*	336.0 ± 125.9		0.01995	3.00
Mullidae	*Parupeneus macronemus*	131.8 ± 26.3	152.5 ± 167.4	0.00912	3.15
Ostraciidae	*Ostracion cubicus*	95.6	42.6	0.05248	2.76
Scaridae	*Calotomus viridescens*	178.3	105.3	0.02089	2.98
	*Cetoscarus bicolor*	164.8		0.01445	3.03
	*Chlorurus sordidus*	377.8 ± 8.5	126.6 ± 96.5	0.01585	3.05
	*Scarus ferrugineus*	254.8 ± 46.7	111.7 ± 72.8	0.01445	3.00
	*Scarus niger*	275.5 ± 81.7	55.6 ± 4.5	0.01622	3.04
Siganidae	*Siganus luridus*	162.7 ± 53.4	39.3 ± 30.9	0.01288	2.96
Tetraodontidae	*Canthigaster cyanospilota*	106.9	68 ± 7.8	0.02818	2.94

**TABLE A2:** Akaike Information Criterion (AIC) values for linear mixed‐effects models examining the relationship between surgeonfish species, individual mass, and either bite rate or bite distance, with varying combinations of Fish identity (ID) and Quadrat ID as random effects. The lowest AIC value is marked in bold.

Independent variables	Dependent variable	Random effects	AIC
Species + Mass	Bite rate	Fish ID + Quadrat ID	200.5431
Species	Bite rate	Fish ID + Quadrat ID	187.8529
Species + Mass	Bite rate	Fish ID	198.5431
Species	Bite rate	Fish ID	185.8529
Species + Mass	Bite rate		185.2544
Species	Bite rate		**183.2562**
Species + Mass	Bite distance	Fish ID + Quadrat ID	218.4714
Species	Bite distance	Fish ID + Quadrat ID	205.2367
Species + Mass	Bite distance	Fish ID	216.4714
Species	Bite distance	Fish ID	203.2367
Species + Mass	Bite distance		196.8243
Species	Bite distance		**195.0681**
